# Adipose tissue endocannabinoid system gene expression: depot differences and effects of diet and exercise

**DOI:** 10.1186/1476-511X-10-194

**Published:** 2011-10-28

**Authors:** Tongjian You, Beth L Disanzo, Xuewen Wang, Rongze Yang, Dawei Gong

**Affiliations:** 1 Department of Exercise and Health Sciences, University of Massachusetts Boston, Boston, MA 02125, USA; 2 Department of Exercise and Nutrition Sciences, State University of New York at Buffalo, Buffalo, NY 14214, USA; 3 Center for Human Nutrition, Division of Geriatrics and Nutritional Science, Washington University School of Medicine, St. Louis, MO 63110, USA; 4 Division of Endocrinology, Diabetes and Nutrition, Department of Medicine, University of Maryland School of Medicine, Baltimore, MD 21201, USA

**Keywords:** Cannabinoid Type 1 Receptor, Fatty Acid Amide Hydrolase, Fat Depots, Diet, Exercise

## Abstract

**Background:**

Alterations of endocannabinoid system in adipose tissue play an important role in lipid regulation and metabolic dysfunction associated with obesity. The purpose of this study was to determine whether gene expression levels of cannabinoid type 1 receptor (CB1) and fatty acid amide hydrolase (FAAH) are different in subcutaneous abdominal and gluteal adipose tissue, and whether hypocaloric diet and aerobic exercise influence subcutaneous adipose tissue CB1 and FAAH gene expression in obese women.

**Methods:**

Thirty overweight or obese, middle-aged women (BMI = 34.3 ± 0.8 kg/m^2^, age = 59 ± 1 years) underwent one of three 20-week weight loss interventions: caloric restriction only (CR, N = 9), caloric restriction plus moderate-intensity aerobic exercise (CRM, 45-50% HRR, N = 13), or caloric restriction plus vigorous-intensity aerobic exercise (CRV, 70-75% HRR, N = 8). Subcutaneous abdominal and gluteal adipose tissue samples were collected before and after the interventions to measure CB1 and FAAH gene expression.

**Results:**

At baseline, FAAH gene expression was higher in abdominal, compared to gluteal adipose tissue (2.08 ± 0.11 vs. 1.78 ± 0.10, expressed as target gene/β-actin mRNA ratio × 10^-3^, P < 0.05). Compared to pre-intervention, CR did not change abdominal, but decreased gluteal CB1 (Δ = -0.82 ± 0.25, P < 0.05) and FAAH (Δ = -0.49 ± 0.14, P < 0.05) gene expression. CRM or CRV alone did not change adipose tissue CB1 and FAAH gene expression. However, combined CRM and CRV (CRM+CRV) decreased abdominal adipose tissue FAAH gene expression (Δ = -0.37 ± 0.18, P < 0.05). The changes in gluteal CB1 and abdominal FAAH gene expression levels in the CR alone and the CRM+CRV group were different (P < 0.05) or tended to be different (P = 0.10).

**Conclusions:**

There are depot differences in subcutaneous adipose tissue endocannabinoid system gene expression in obese individuals. Aerobic exercise training may preferentially modulate abdominal adipose tissue endocannabinoid-related gene expression during dietary weight loss.

**Trial Registration:**

ClinicalTrials.gov: NCT00664729.

## Background

Obesity is a risk factor for the development of type 2 diabetes and cardiovascular disease [1,2]. Endocannabinoids, which are derived from membrane phospholipids (anandamide [AEA]) and triglycerides (2-arachidonoylglycerol [2-AG]), increase food intake and weight gain by activating central endocannabinoid receptors [3,4]. With obesity, endocannabinoid dysregulation occurs in peripheral sites, linking the enlarged adipose tissue and metabolic dysfunction [4-7].

In rodents, endocannabinoids bind to G-protein-coupled cannabinoid type-1 (CB1) and cannabinoid type-2 (CB2) receptors. CB1 receptors are mainly expressed in the brain, digestive organs and adipose tissue, whereas CB2 receptors are mainly expressed in immune cells [8]. Endocannabinoids are degraded by the key enzyme fatty acid amide hydrolase (FAAH) in these tissue/cells [9]. In overweight and obese subjects, depot differences in adipose tissue CB1 and FAAH gene expression levels were compared in several studies. Some [10,11], but not all [12] studies, support that adipose tissue gene expression levels of CB1 and FAAH are higher in visceral than in abdominal subcutaneous fat. However, it is important to study if gene expression of these important components of the endocannabinoid system are different between abdominal and gluteal subcutaneous fat tissue.

Current studies indicate that weight loss by 5% does not change adipose tissue gene expression levels of CB1 and FAAH in abdominal subcutaneous adipose tissue [7,13]. However, it is not known if substantial weight loss (> 10%) by diet and exercise could change gene expression of CB1 and FAAH in adipose tissue. Moreover, although acute exercise activates the endocannabinoid system [14], the effect of aerobic exercise training on endocannabinoid-related gene expression in adipose tissue is yet to be investigated.

Thus, we tested the hypothesis that there is depot difference in subcutaneous adipose tissue CB1 and FAAH gene expression, and caloric restriction and aerobic exercise training alter subcutaneous adipose tissue CB1 and FAAH gene expression in obese older women.

## Methods

### Subjects

All women were recruited according to the following inclusion/exclusion criteria: 1) overweight or obese (BMI = 25-40 kg/m^2 ^and waist girth > 88 cm), 2) older (age = 50-70 yrs, and at least one year without menses), 3) non-smoking, 4) not on hormone replacement therapy, 5) sedentary (< 15 min of exercise, 2 times/wk) in the past 6 months, and 6) weight-stable (< 5% weight change) for at least 6 months prior to enrollment. The study was approved by the Wake Forest University Institutional Review Board for Human Research. All women signed informed consent to participate in the study.

Women with evidence of untreated hypertension (blood pressure > 160/90 mmHg), hypertriglyceridemia (triglycerides > 400 mg/dl), insulin-dependent diabetes, active cancer, liver, renal or hematological disease were excluded after an initial screening included a medical history review, physical examination, fasting blood profile (lipoprotein lipids and glucose) and 12-lead resting electrocardiogram. In addition, all subjects underwent a graded treadmill exercise test to exclude those with an abnormal cardiovascular response to exercise [15]. All women were randomly assigned to either a caloric restriction alone (CR), CR plus moderate-intensity exercise (CRM), or CR plus vigorous-intensity exercise (CRV) intervention for a period of 20 weeks.

### Study design

This study was part of the Diet, Exercise, and Metabolism for Older Women Study, a randomized completed from 2003 to 2007 [16-19]). Baseline measurements of body composition, maximal aerobic capacity (VO_2_max), and adipose tissue CB1 and FAAH gene expression were performed after at least 2 weeks of weight stability before the interventions. Body composition and VO_2_max were measured on the same day. Fat biopsies were performed in a morning after an over-night fast, and at least 5 days after the VO_2_max test. The women were retested in the same manner after the 20-week interventions. The post-intervention fat biopsies occurred at least 2 days after the last exercise session.

### Study interventions

During the 20-week interventions, all women were provided food for their lunch and supper, which was prepared by the Wake Forest University General Clinical Research Center (GCRC) metabolic kitchen staff. These meals were prepared individually after women chose from a hypocaloric menu designed by a registered dietitian (RD). Women purchased and prepared their breakfast meal, in consultation with the RD. They were allowed 2 free days per month, during which they were given guidelines for diet intake and asked to report all intake. They were also allowed to consume as many non-caloric, non-caffeinated beverages as they liked. In addition, all women were provided with a daily calcium supplement (1000 mg/day).

The diet only group was asked not to alter their physical activity habits during the study. Both diet plus exercise groups walked on a treadmill three days/week at a target heart rate calculated from the Karvonen equation [(HRR × (intensity)+ resting heart rate] [20], where heart rate reserve (HRR) is maximal heart rate minus resting heart rate obtained from each subject's VO_2_max test. The duration and intensity of the exercise progressed from 15-20 min at 45-50% of HRR during the first week to 55 min at 45-50% HRR for the moderate-intensity group, and 30 min at 70-75% HRR for the vigorous-intensity group by the second month. The calorie deficits of all women were adjusted to ~2800 kcal/week. The deficits for the diet only group resulted totally from reduction in dietary intake, whereas deficits for the diet plus exerciser groups resulted from both reductions in dietary intake (~2400 kcal/week) and in exercise expenditure (~400 kcal/week). The average daily calorie intake recorded by all women was 100.3 ± 0.2% of the provided calorie level. The exercise compliance (attendance at scheduled sessions) was 81.0 ± 8.9% for the moderate-intensity exercise group, and 85.4 ± 9.3% for the vigorous-intensity exercise group.

### Body composition

Height and weight of each woman were measured to calculate BMI (kg/m^2^). Waist (minimal circumference) was measured by a tape measure. Fat mass, lean mass and percent body fat were measured by dual energy X-ray absorptiometry (Hologic Delphi QDR, Bedford, MA).

### Maximal aerobic capacity

VO_2_max was measured on a motor-driven treadmill (Medical Graphics Corporation, Minneapolis, MN) during a graded exercise test. A ramp treadmill protocol was used. Each test was set for a duration of 12 minutes with a goal of 12 metabolic equivalents, and the treadmill self-adjusted the incline to reach that goal. A valid VO_2_max was obtained when a respiratory exchange ratio (RER) of 1.10 had been reached. If the participant did not reach this criterion, the test was repeated.

### Adipose tissue gene expression

Subcutaneous abdominal and gluteal adipose tissue was taken by aspiration with a 16-gauge needle under local anesthesia (2% xylocaine) after an overnight fast. The samples were put in warm saline and transported immediately to the laboratory where they were washed twice with saline to eliminate blood and other connective tissue. Immediately after the washing, approximately 0.5 g of tissue was snap frozen in liquid nitrogen and then stored at -80°C for later isolation of total RNA for CB1, FAAH, IL-6 and TNF-α gene expression.

Total RNA was isolated from frozen adipose tissue samples with the RNeasy lipid tissue kit (Qiagen, Valencia, CA). The isolated total RNA was quantified by measurement of absorbency at 260 and 280 nm, and its integrity was verified using agarose gels (1%) stained with ethidium bromide. Total RNA samples were stored at -80°C until measurement of gene expression.

CB1, FAAH, IL-6 and TNF-α mRNA expression levels were measured by real-time RT-PCR. First, 1 μg of total RNA was used for the reverse transcription reaction to synthesize the first-strand cDNA, using the random hexamer primers and following the instructions of the Advantage RT-for-PCR Kit (Clontech, Palo Alto, CA). Real-time quantification of target gene (CB1, FAAH, IL-6 and TNF-α) to β-actin mRNA was performed, using ABI Taqman PCR kits on an ABI PRISM 7900 Sequence Detection System (Applied Biosystems, Foster City, CA). CB1, FAAH, IL-6, TNF-α and β-actin mRNA were amplified in different wells and in duplicates, and the increase in fluorescence was measured in real time. Data were obtained as threshold cycle (C_T_) values. Relative gene expression was calculated using the formula (1/2) ^CT Target Gene-CT β-actin^.

### Statistical analyses

Statistical analyses were performed using IBM SPSS Statistics 19 (Armonk, NY). First, general characteristics of study participants were compared using one-way *ANOVA *(for continuous variables) or the Chi-square test (for categorical variables). Differences in body composition, aerobic fitness, and metabolic variables among the intervention groups (CR, CRM and CRV) at baseline and over-time changes in response to the interventions were determined using one-way *ANOVA*. The LSD *post-hoc *test was used to determine any group differences if an overall group effect was ascertained. Depot (gluteal and abdominal) differences in adipose tissue gene expression at baseline in the whole cohort, and the within-group differences between pre-intervention and post-intervention measures of all variables were determined using a paired *t*-test. Differences in CB1, FAAH, IL-6 and TNF-α gene expression among the intervention groups (CR, CRM and CRV) at baseline and over-time changes were determined using one-way *ANOVA*. The LSD *post-hoc *test was used for identifying any group differences. The differences in CB1 and FAAH gene expression between the diet alone (CR) and combined diet plus exercise groups (CRM+CRV) were also determined by using a paired t-test. Correlations between CB1 or FAAH and cytokines were determined by using Pearson parametric or Spearman nonparametric analysis. All data are presented as mean ± standard error, and the level of significance was set at P < 0.05 for all analyses.

## Results

### Subject characteristics

Thirty women (CR: N = 9; CRM: N = 13; CRV: N = 8) completed the intervention and had sufficient adipose tissue sample amounts for analysis on both fat depots. General characteristics of these 30 women are shown in Table 1 by intervention group. There were no group differences in age, years post-menopause, or percent of African Americans.

**Table 1:** General characteristics of the study participants.

	CR (n = 9)	CRM (n = 13)	CRV (n = 8)
Age (yrs)	59 ± 2	57 ± 2	62 ± 2
Post-menopause (yrs)	15 ± 2	11 ± 3	20 ± 6
African American (%)	11.1	30.8	12.5

Mean ± SE or %. General characteristics of study participants were compared using one-way *ANOVA *(for continuous variables) or the Chi-square test (for categorical variables). CR: caloric restriction only; CRM: caloric restriction plus moderate-intensity aerobic exercise; CRV: caloric restriction plus vigorous-intensity aerobic exercise.

### Effects of caloric restriction alone, caloric restriction plus moderate-intensity exercise, and caloric restriction plus vigorous-intensity exercise on body composition, aerobic fitness, and metabolic variables

Body composition and aerobic fitness measures before and after the interventions in all 3 groups are shown in Table 2. At baseline, there were no group differences in body weight, fat mass, percent body fat, waist, maximal aerobic capacity (absolute and relative VO_2_max), and fasting glucose levels. However, there were significant higher insulin levels in CRV compared to CR and CRM groups (both P < 0.01). All three interventions reduced body weight, fat mass, percent body fat, and waist (all P < 0.01). All three interventions did not change absolute VO_2_max; however, CRM and CRV, but not CR alone, increased relative VO_2_max (both P < 0.01). While only CRV reduced fasting glucose levels (P < 0.05), all three interventions decreased fasting insulin (P < 0.05 to P < 0.01). All three groups lost a similar amount of body weight (CR: -11.1 ± 1.3%; CRM: -13.3 ± 0.9%; CRV: -13.8 ± 1.6%). Compared to CR alone, CRV resulted in more reductions in fat mass, percent body fat, fasting insulin and HOMA index (all P < 0.05). There were no differences in reductions in waist circumference, maximal aerobic capacity and fasting glucose in all three groups.

**Table 2:** Body composition, maximal aerobic capacity, and metabolic variables before and after interventions.

	CR (n = 9)		CRM (n = 13)		CRV (n = 8)	
	Pre	Post	Pre	Post	Pre	Post
Weight (kg)	86.8 ± 3.6	75.7 ± 3.8^†^	93.8 ± 2.9	80.5 ± 2.6^†^	92.4 ± 5.5	78.5 ± 4.7^†^
Fat mass (kg)	37.3 ± 2.4	30.2 ± 2.8^†^	42.3 ± 1.5	32.8 ± 1.2^†^	43.0 ± 3.1	31.6 ± 2.6^†^
Percent fat (%)	41.7 ± 1.1	38.6 ± 1.7^†^	44.1 ± 0.9	40.0 ± 0.9^†^	45.0 ± 0.8	39.2 ± 1.2^†^
Waist (cm)	95 ± 3	86 ± 3^†^	98 ± 2	87 ± 2^†^	100 ± 3	89 ± 3^†^
Absolute VO_2_max (l/min)	1.87 ± 0.09	1.82 ± 0.12	1.96 ± 0.08	1.97 ± 0.07	1.90 ± 0.19	2.16 ± 0.14
Relative VO_2_max (ml/min/kg)	21.6 ± 0.8	23.6 ± 1.4	21.5 ± 1.0	24.2 ± 0.8*	20.3 ± 1.4	26.2 ± 1.0*
Glucose (mg/dl)	95.4 ± 2.9	90.7 ± 1.4	94.0 ± 2.4	90.2 ± 2.0	102.1 ± 6.9	91.3 ± 3.6*
Insulin (pmol/l)	91.5 ± 12.1	70.2 ± 12.5*	66.4 ± 10.8	43.7 ± 6.7*	147 ± 26.1	83.5 ± 12.3^†^

Mean ± SE. Body composition, maximal aerobic capacity, and metabolic variables were compared using paired tests. VO2max: maximal aerobic capacity; HOMA: homeostatic model assessment. *P < 0.05, ^† ^P < 0.01 compared with baseline.

### Effects of caloric restriction alone, caloric restriction plus moderate-intensity exercise, and caloric restriction plus vigorous-intensity exercise on adipose tissue endocannabinoid system gene expression

**Figure 1:**
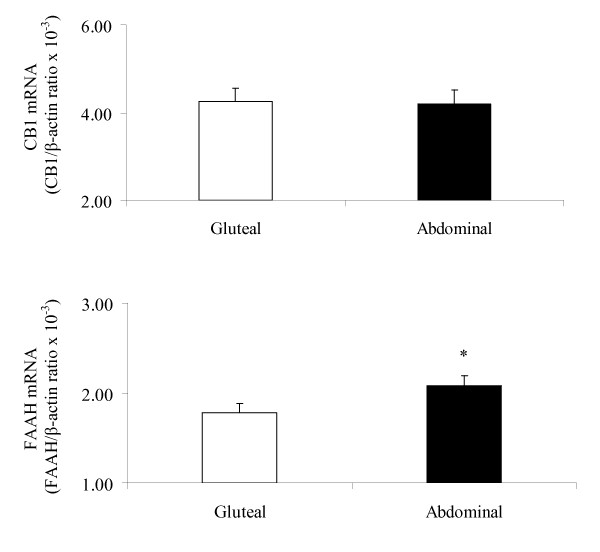
**Depot differences in adipose tissue CB1 and FAAH gene expression (mean ± SE, n = 30)**. Gene expression levels of CB1 and FAAH between gluteal and abdominal adipose tissue depots were compared using paired tests. *P < 0.05 compared to Gluteal.

At baseline, FAAH gene expression was higher in subcutaneous abdominal, compared to gluteal adipose tissue (P < 0.05), whereas there was no depot difference in adipose tissue CB1 gene expression (Figure 1).

Adipose tissue CB1 and FAAH gene expression levels before and after the interventions in all 3 groups are shown in Table 3. At baseline, there were no group differences in adipose tissue CB1 and FAAH mRNA levels. Compared to pre-intervention, CR alone did not change abdominal, but decreased gluteal CB1 and FAAH gene expression (both P < 0.05). CRM or CRV alone did not change abdominal and gluteal adipose tissue CB1 and FAAH gene expression.

**Table 3:** Adipose tissue CB1 and FAAH gene expression before and after interventions.

	CR (n = 9)		CRM (n = 13)		CRV (n = 8)	
	Pre	Post	Pre	Post	Pre	Post
CB1/β-actin mRNA ratio (× 10^-3^)						
Abdominal	4.22 ± 0.78	5.40 ± 0.10	3.94 ± 0.36	3.71 ± 0.29	4.59 ± 0.56	4.41 ± 0.69
Gluteal	4.84 ± 0.55	4.02 ± 0.66*	3.74 ± 0.28	3.99 ± 0.42	4.43 ± 0.86	4.83 ± 0.72
FAAH/β-actin mRNA ratio (× 10^-3^)						
Abdominal	1.86 ± 0.20	2.05 ± 0.24	2.32 ± 0.19	1.94 ± 0.16	1.93 ± 0.15	1.59 ± 0.17
Gluteal	1.82 ± 0.21	1.33 ± 0.19*	1.86 ± 0.15	1.70 ± 0.14	1.60 ± 0.17	1.36 ± 0.13

Mean ± SE. Gene expression levels were compared using paired tests. *P < 0.05 compared with baseline.

However, combined CRM and CRV groups decreased abdominal adipose tissue FAAH gene expression (P < 0.05). CRM+CRV did not change gluteal adipose tissue FAAH gene expression, or gluteal and abdominal adipose tissue CB1 gene expression.

**Figure 2:**
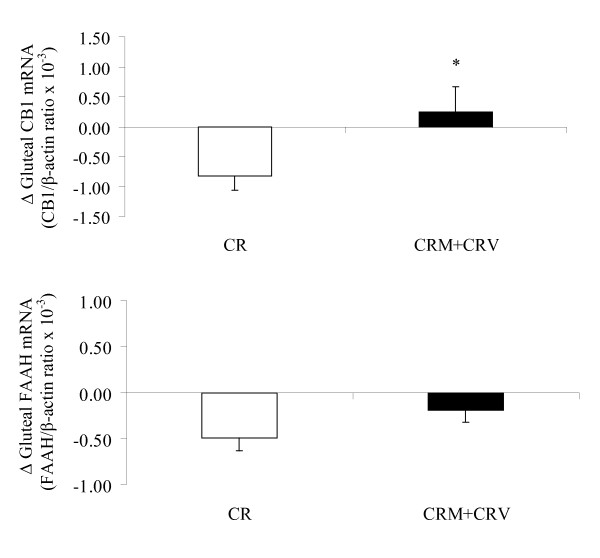
**Effects of caloric restriction and caloric restriction plus exercise on gluteal adipose tissue CB1 and FAAH gene expression (mean ± SE, CR: n = 9; CRM+CRV: n = 21)**. Changes in gene expression levels of CB1 and FAAH between gluteal and abdominal adipose tissue depots were compared between caloric restriction and caloric restriction plus aerobic exercise using paired tests. *P < 0.05 compared to CR.

**Figure 3:**
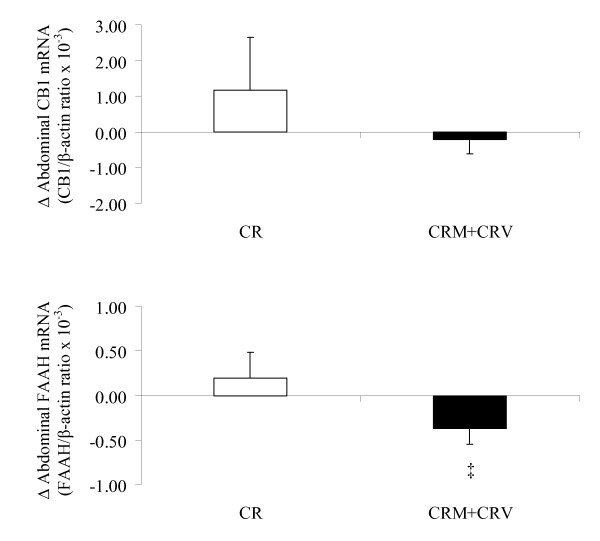
**Effects of caloric restriction and caloric restriction plus exercise on abdominal adipose tissue CB1 and FAAH gene expression (mean ± SE, CR: n = 9; CRM+CRV: n = 21)**. Changes in gene expression levels of CB1 and FAAH between gluteal and abdominal adipose tissue depots were compared between caloric restriction and caloric restriction plus aerobic exercise using paired tests. ^‡ ^P = 0.10 compared to CR.

The changes in gluteal adipose tissue CB1 gene expression levels in the CR alone group were different from those in the CRM+CRV groups (P < 0.05), whereas there was no group difference in gluteal adipose tissue FAAH gene expression (Figure 2). The changes in abdominal adipose tissue FAAH gene expression levels in the CRM+CRV groups tended to be different from those in the CR group (P = 0.10), whereas there was no group difference in abdominal adipose tissue CB1 gene expression (Figure 3).

### Effects of caloric restriction alone, caloric restriction plus moderate-intensity exercise, and caloric restriction plus vigorous-intensity exercise on adipose tissue cytokine gene expression

At baseline, IL-6 gene expression was higher in subcutaneous gluteal, compared to abdominal adipose tissue (4.35 ± 0.57 vs. 1.96 ± 0.28, expressed as target gene/β-actin mRNA ratio × 10^-2^, P < 0.01), whereas there was no depot difference in adipose tissue TNF-α gene expression.

Adipose tissue IL-6 and TNF-α gene expression levels before and after the interventions in all 3 groups are shown in Table 4. At baseline, there were no group differences in adipose tissue IL-6 and TNF-α mRNA levels. Compared to pre-intervention, CR, CRM or CRV alone did not change abdominal and gluteal adipose tissue IL-6 and TNF-α gene expression. However, in the whole cohort, combined diet and exercise interventions significantly reduced gluteal adipose tissue gene expression levels of IL-6 (from 4.35 ± 0.57 to 2.50 ± 0.40, P < 0.05) and TNF-α (from 4.75 ± 1.16 to 2.58 ± 0.44, P < 0.05).

**Table 4:** Adipose tissue IL-6 and TNF-α gene expression before and after interventions.

	CR (n = 9)		CRM (n = 13)		CRV (n = 8)	
	Pre	Post	Pre	Post	Pre	Post
IL-6/β-actin mRNA ratio (× 10^-2^)						
Abdominal	2.37 ± 0.66	1.47 ± 0.50	1.65 ± 0.32	2.09 ± 0.78	2.15 ± 0.44	2.20 ± 0.80
Gluteal	4.29 ± 0.83	1.75 ± 0.69	4.87 ± 1.00	2.66 ± 0.31	3.63 ± 1.19	2.92 ± 1.12
TNF-α/β-actin mRNA ratio (× 10^-2^)						
Abdominal	4.46 ± 2.20	3.09 ± 1.22	3.62 ± 1.55	2.12 ± 0.39	4.11 ± 2.37	2.48 ± 0.69
Gluteal	5.19 ± 2.11	2.09 ± 0.51	5.63 ± 2.29	2.81 ± 0.55	2.90 ± 0.81	2.84 ± 1.35

Mean ± SE. Gene expression levels were compared using paired tests.

### Relationship of adipose tissue endocannabinoid system gene expression to cytokine gene expression after diet and exercise interventions

In the whole cohort, change in abdominal CB1 gene expression tended to be related to change in abdominal IL-6 gene expression (r = 0.33, P = 0.07), and change in gluteal FAAH gene expression tended to be related to change in gluteal TNF-gene expression (r = 0.31, P = 0.09). No other significant relationships were found between changes in local adipose tissue CB1 or FAAH and cytokine gene expression levels.

## Discussion

This study investigated whether there was a depot difference in abdominal and gluteal subcutaneous adipose tissue gene expression of two important components of the endocannabinoid system, CB1 and FAAH, and whether caloric restriction alone, and caloric restriction plus aerobic exercise differentially influenced adipose tissue CB1 and FAAH gene expression in obese older women. Our findings showed that compared to gluteal adipose tissue, abdominal adipose tissue had higher FAAH gene expression. Caloric restriction alone lowered gluteal CB1 and FAAH gene expression, whereas caloric restriction plus aerobic exercise reduced abdominal adipose tissue FAAH gene expression.

Previous studies indicate that circulating levels of endocannabinoids are elevated in obesity, possibly reflecting a partial overflow from the tissues that produce and release endocannabinoids [21]. Adipose tissue expresses all the essential components of the endocannabinoid system, therefore is possible the main organ that contributes to the elevated circulating endocannabinoid levels and metabolic dysfunctions [22]. The increased adipose tissue endocannabinoid levels could be due to an increased production and/or a decreased degradation. Several human studies reported that CB1 and FAAH gene expression in visceral and subcutaneous adipose tissue were decreased with obesity and the expression levels were negatively related to visceral fatness [7,10,23]. However, other studies reported no changes in CB1 gene expression in visceral and subcutaneous adipose tissue [12], or even elevated CB1 and FAAH gene expression in visceral and subcutaneous adipose tissue with obesity [24,25]. Two recent studies indicated that CB1 gene expression in visceral adipose tissue was positively related to visceral fat mass in obese-hypertensive patients [26], and FAAH activity in subcutaneous adipose tissue correlated positively to BMI in healthy adults [27]. All these studies were cross-sectional comparison studies.

Several previous human studies compared the depot differences in adipose tissue CB1 and FAAH gene expression. Interestingly, some studies indicate that visceral adipose tissue expresses higher levels of CB1 and FAAH than subcutaneous adipose tissue in obese individuals [10,11]. In one study, although obese subjects had lower expression of CB1 and FAAH in both subcutaneous and visceral adipose tissue than lean subjects, CB1 and FAAH expression levels in visceral adipose tissue are higher than those in subcutaneous adipose tissue in all three groups, including lean, subcutaneous obese and visceral obese individuals [10]. A recent study reported that there were depot differences in subcutaneous adipose tissue CB1 gene expression levels in obese individuals [28]. Here, our findings support that there are depot differences in upper and lower body subcutaneous adipose tissue FAAH gene expression in obese women. The differences in FAAH expression among different adipose tissue depots could be due to the differences in endocannabinoid levels in these depots, which are influenced by adipose tissue inflammatory cytokines.

There are limited data regarding the effects of weigh loss and/or exercise training on adipose tissue endocannabinoid system in obesity. In two previous studies, weight loss by 5% did not change abdominal adipose tissue CB1 and FAAH gene expression [7,13]. Weight loss also did not influence circulating levels of endocannabinoids [7,13]. In a recent study, dietary weight loss by 10-12% altered subcutaneous adipose tissue 2-AG levels and lowered CB1 and FAAH gene expression levels in gluteal, but not abdominal adipose tissue [28]. The possible reason for the mixed results could be the differences in the amounts of weight loss in these studies. In an animal study, high-fat feeding significantly increased visceral and subcutaneous adipose tissue CB1 protein expression and exercise training inhibited this high-fat feeding related effects on adipose tissue CB1 protein expression [29]. However, there were no human studies regarding the effects of exercise training on adipose tissue endocannabinoid system.

In the current study, we observed that caloric restriction lowered gluteal adipose tissue CB1 and FAAH gene expression. These results support the findings of another study [28] and may indicate that weight loss by more than 10% may alter adipose tissue endocannabinoid-related gene expression. Moreover, caloric restriction plus exercise training, including moderate- and vigorous-intensity aerobic exercise, lowered abdominal adipose tissue FAAH gene expression. In a previous study, abdominal adipose tissue FAAH gene expression is positively related to hyerinsulinemia [25]. Moreover, visceral adipose tissue endocannabinoid system activation was mediated by inflammatory cytokines in obesity [23]. In the current study, changes in endocannabinoid system gene expression tended to be related to changes in IL-6 and TNF-α gene expression, indicating a possible link between endocannabinoid system and chronic inflammation in obese individual under diet and exercise interventions.

The findings that abdominal adipose tissue expresses higher levels of FAAH than gluteal adipose tissue, and caloric restriction and caloric restriction plus exercise differentially influenced local adipose tissue endocannabinoid system are interesting. It is notable that circulating and adipose tissue levels of AEA and 2-AG were not measured. Addition of these measures in future studies will provide us with a clearer picture of the depot differences and intervention effects on adipose tissue endocannabinoid system. One of the limitations of the current study is that the sample size in each group was relatively small. With bigger sample size, we would be able to detect a significant exercise effect on abdominal adipose tissue FAAH gene expression in obese individuals under caloric restriction.

## Conclusions

Subcutaneous adipose tissue FAAH gene expression is higher in abdominal fat depot than in gluteal fat depot; caloric restriction alone altered gluteal adipose tissue CB1 and FAAH gene expression, whereas caloric restriction plus aerobic exercise changed abdominal adipose tissue FAAH gene expression. These effects may be of importance for the metabolic properties of local adipose tissue and the effects of diet and exercise interventions on adipose tissue metabolic function. Since the studies on adipose tissue endocannabinoid system are on the early stages and mixed findings exist in these studies, further investigations need to focus on the relationship of the components of this system to local and system metabolic risk factors in future cross-sectional or intervention studies.

## List of Abbreviations

2-AG: 2-arachidonoylglycerol; AEA: anandamide; BMI: body mass index; CR: caloric restriction; CB1: cannabinoid type 1 receptor; CRM: caloric restriction plus moderate-intensity aerobic exercise; CRV: caloric restriction plus vigorous-intensity aerobic exercise; FAAH: fatty acid amide hydrolase; HOMA: homeostatic model assessment; HRR: heart rate reserve; RER: respiratory exchange ratio; RT-PCR: reverse transcription-polymerase chain reaction; VO2max: maximal aerobic capacity.

## Competing interests

The authors declare that they have no competing interests.

## Authors' contributions

TY study design and supervision, data collection and analysis, manuscript writing. BLD data interpretation and manuscript writing. XW study supervision and data collection. RY data collection. DG study concept and design, data collection. All authors have read, edited and approved the final manuscript.
